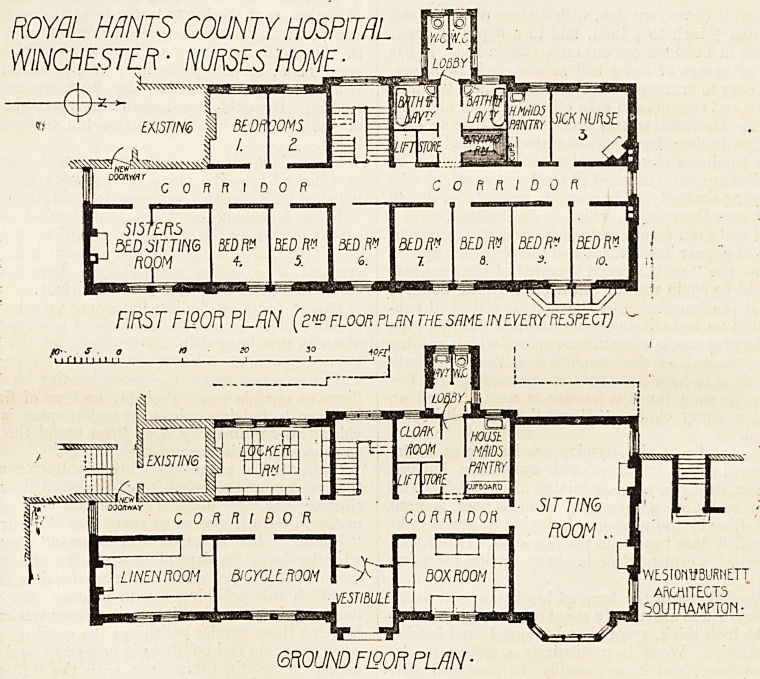# Royal Hants County Hospital, Winchester—Nurses' Home

**Published:** 1911-08-26

**Authors:** 


### ROYAL HANTS COUNTY HOSPITAL, WINCHESTER—NURSES' HOME.

This building is an extension of the existing hospital to the north, and provides accommodation for two sisters, eighteen nurses, and two sick-rooms.

On the ground floor is the main entrance from the outside, to the left of which is a bicycle-room, entered from the vestibule and also from the corridor.

On the right is a box-room and a large sitting-room, 29 feet 6 inches by 18 feet 3 inches wide. Adjoining the bicycle-room is a linen room, and at the back is a locker-room, housemaids' pantry, a very small not provided with fireplaces, and there is no indication of any method of ventilation other than windows.

The arrangement for the sisters seems somewhat peculiar. It is practically two rooms with a large opening connecting them and one door from the corridor giving access to the whole room. Obviously the part used as sitting-room would be the part where the fireplace is, and it is difficult to see any good reason for omitting the door from the bed-room part into the corridor, or for making the wide opening between the two. A much better arrangement would have cloak-room, a lift, and a store cupboard. A projecting sanitary tower contains a w.c. and a lavatory, and has a separate entrance from the outside. The provision of one large sitting-room for all the nurses is generally found to be a mistake. As a matter of fact, the nurses do not use their sitting-room to a very large extent, and it is better to provide one room where a nurse can read or write in quiet and another room where conversation and music can go on without annoyance to those who wish to be quiet.

The two upper floors are exactly similar, and each contains nine bedrooms for nurses, a bed-sitting-room for a sister, a sick room for nurses with a housemaids' pantry adjoining, a drying room, two bath-rooms, and two w.c.s, besides a lift and a store cupboard. The bedrooms are been to have made two separate rooms and have entered the bedroom from the corridor in the usual way.

The bath-rooms are certainly very restricted in size, and if the baths are fixed as shown on the plans there is no possibility of cleaning at the back of them. A suitable slop sink and housemaids' closet would appear to be needed.

The building was erected from the designs of Messrs. Weston and Burnett, of Southampton.

## Figures and Tables

**Figure f1:**